# *catena*-Poly[silver(I)-μ-acridine-9-carboxyl­ato-κ^3^ *N*:*O*,*O*′]

**DOI:** 10.1107/S1600536810043199

**Published:** 2010-10-30

**Authors:** Ya-Qing Yang, Xiao-Ye Chen, Shu-Min Huo, Yan-Ru Ma, Rong-Hua Zeng

**Affiliations:** a School of Chemistry and Environment, South China Normal University, Guangzhou 510006, People’s Republic of China; b Key Laboratory of Technology on Electrochemical Energy Storage and Power Generation in Guangdong Universities, South China Normal University, Guangzhou 510006, People’s Republic of China

## Abstract

In the title coordination polymer, [Ag(C_14_H_8_NO_2_)]_*n*_, the Ag^I^ cation is coordinated by two O atoms and one N atom from two symmetry-related acridine-9-carboxyl­ate ligands in a distorted trigonal-planar geometry. The metal atoms are connected by the ligands to form chains running parallel to the *b* axis. π–π stacking inter­actions [centroid-to-centroid distances 3.757 (2)–3.820 (2) Å] and weak Ag⋯O inter­actions further link the chains to form a layer network parallel to the *ab* plane. The Ag^I^ cation is disordered over two positions, with refined site-occupancy factors of 0.73 (3):0.27 (3).

## Related literature

For the structures of related metal complexes of acridine-9-carboxyl­ate, see: Bu, Tong, Chang *et al.* (2005 ▶); Bu, Tong, Li *et al.* (2005 ▶); Bu, Tong, Xie *et al.* (2005 ▶).
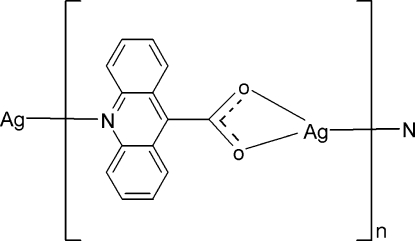


## Experimental

### 

#### Crystal data

[Ag(C_14_H_8_NO_2_)]
*M* *_r_* = 330.08
Monoclinic, 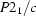

*a* = 7.5622 (7) Å
*b* = 9.2210 (9) Å
*c* = 16.4451 (14) Å
β = 111.494 (4)°
*V* = 1066.99 (17) Å^3^
*Z* = 4
Mo *K*α radiation
μ = 1.88 mm^−1^
*T* = 273 K
0.22 × 0.19 × 0.17 mm

#### Data collection

Bruker APEXII area-detector diffractometer
Absorption correction: multi-scan (*SADABS*; Sheldrick, 2008 ▶) *T*_min_ = 0.683, *T*_max_ = 0.741
5598 measured reflections
2084 independent reflections
1567 reflections with *I* > 2σ(*I*)
*R*_int_ = 0.023

#### Refinement

*R*[*F* ^2^ > 2σ(*F* ^2^)] = 0.029
*wR*(*F* ^2^) = 0.077
*S* = 1.06
2084 reflections
173 parameters
H-atom parameters constrained
Δρ_max_ = 0.54 e Å^−3^
Δρ_min_ = −0.30 e Å^−3^

### 

Data collection: *APEX2* (Bruker, 2004 ▶); cell refinement: *SAINT* (Bruker, 2004 ▶); data reduction: *SAINT*; program(s) used to solve structure: *SHELXS97* (Sheldrick, 2008 ▶); program(s) used to refine structure: *SHELXL97* (Sheldrick, 2008 ▶); molecular graphics: *SHELXTL* (Sheldrick, 2008 ▶); software used to prepare material for publication: *SHELXTL*.

## Supplementary Material

Crystal structure: contains datablocks I, global. DOI: 10.1107/S1600536810043199/rz2505sup1.cif

Structure factors: contains datablocks I. DOI: 10.1107/S1600536810043199/rz2505Isup2.hkl

Additional supplementary materials:  crystallographic information; 3D view; checkCIF report

## Figures and Tables

**Fig. 1.**
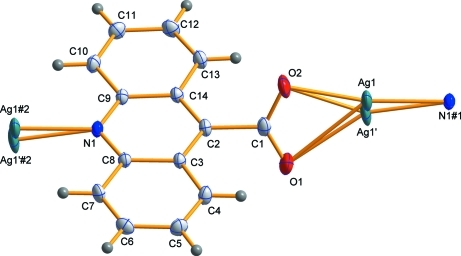
The molecular structure showing the atomic-numbering scheme. Displacement ellipsoids drawn at the 30% probability level. Symmetry codes: (#1) x, -1 + y, z; (#2) x, 1 + y, z.

**Fig. 2.**
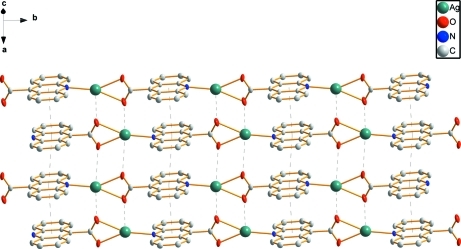
A view of the two-dimensional layer network. π–π stacking and Ag···O interactions are shown as dashed lines.

## supplementary crystallographic information

### Comment

In the synthesis of novel metal organic frameworks (MOFs), ligands play a key role in the construction of coordination polymers with fascinating topologies, intriguing architectures and useful physical-chemical properties. The acridine-9-carboxylate anion is a potential bifunctional ligand with carboxylate and N-donor functional groups which has been used to prepare metal organic complexes possessing multidimensional networks and interesting properties (Bu, Tong, Chang *et al.*, 2005; Bu, Tong, Li *et al.*, 2005; Bu, Tong, Xie *et al.*, 2005). Herein, we report the crystal structure of a novel polymeric silver(I) complex synthesized by the hydrothermal reaction of AgNO_3_ with acridine-9-carboxylic acid in aqueous solution.

As shown in Fig. 1, the asymmetric unit of the title compound consists of a disordered silver(I) ion and one acridine-9-carboxylate anion. The cation is three-coordinated in a distorted trigonal planar geometry by two O atoms and one N atom from two symmetry-related acridine-9-carboxylate ligands. The Ag···O and Ag···N bond lengths range from 2.158 (4) to 2.499 (4) Å and bond angles vary from 55.02 (2) to 154.1 (2)°. The acridine-9-carboxylate ligands connect the metal centres to generate chains parallel to the *b* axis. The chains are further connected by π–π stacking interactions (the centroid-to-centroid distances between neighbouring phenyl rings are 3.757 (2) and 3.820 (2) Å) and Ag···O weak interactions (2.844 (15)–3.348 (16) Å) to assemble a two-dimensional layer network parallel to the *ab* plane (Fig. 2).

### Experimental

A mixture of AgNO_3_ (0.170 g, 1 mmol), acridine-9-carboxylic acid (0.223 g, 1 mmol) and water (10 ml) was stirred vigorously for 60 min and then sealed in a Teflon-lined stainless-steel autoclave (20 ml capacity). The autoclave was heated and maintained at 423 K for 3 d, and then cooled to room temperature at 5 K h^-1^ and obtained the colourless block crystals.

### Refinement

The disordered silver ion was refined over two sites, with refined occupancies of 0.73 (3) and 0.27 (3). H atoms attached to C atoms were placed at calculated positions and were treated as riding on their parent atoms with C—H = 0.93 Å, and with *U*_iso_(H) = 1.2*U*_eq_(C).

### Crystal data

**Table:**

[Ag(C_14_H_8_NO_2_)]	*F*(000) = 648
*M**_r_* = 330.08	*D*_x_ = 2.055 Mg m^−^^3^
Monoclinic, *P*2_1_/*c*	Mo *K*α radiation, λ = 0.71073 Å
Hall symbol: -P 2ybc	Cell parameters from 1873 reflections
*a* = 7.5622 (7) Å	θ = 2.6–27.3°
*b* = 9.2210 (9) Å	µ = 1.88 mm^−^^1^
*c* = 16.4451 (14) Å	*T* = 273 K
β = 111.494 (4)°	Block, colourless
*V* = 1066.99 (17) Å^3^	0.22 × 0.19 × 0.17 mm
*Z* = 4	

### Data collection

**Table:**

Bruker APEXII area-detector diffractometer	2084 independent reflections
Radiation source: fine-focus sealed tube	1567 reflections with *I* > 2σ(*I*)
graphite	*R*_int_ = 0.023
φ and ω scan	θ_max_ = 26.0°, θ_min_ = 2.6°
Absorption correction: multi-scan (*SADABS*; Sheldrick, 2008)	*h* = −9→9
*T*_min_ = 0.683, *T*_max_ = 0.741	*k* = −8→11
5598 measured reflections	*l* = −20→15

### Refinement

**Table:**

Refinement on *F*^2^	Primary atom site location: structure-invariant direct methods
Least-squares matrix: full	Secondary atom site location: difference Fourier map
*R*[*F*^2^ > 2σ(*F*^2^)] = 0.029	Hydrogen site location: inferred from neighbouring sites
*wR*(*F*^2^) = 0.077	H-atom parameters constrained
*S* = 1.06	*w* = 1/[σ^2^(*F*_o_^2^) + (0.0359*P*)^2^ + 0.2862*P*] where *P* = (*F*_o_^2^ + 2*F*_c_^2^)/3
2084 reflections	(Δ/σ)_max_ = 0.006
173 parameters	Δρ_max_ = 0.54 e Å^−^^3^
0 restraints	Δρ_min_ = −0.29 e Å^−^^3^

### Special details

**Table:**

Geometry. All esds (except the esd in the dihedral angle between two l.s. planes) are estimated using the full covariance matrix. The cell esds are taken into account individually in the estimation of esds in distances, angles and torsion angles; correlations between esds in cell parameters are only used when they are defined by crystal symmetry. An approximate (isotropic) treatment of cell esds is used for estimating esds involving l.s. planes.
Refinement. Refinement of F^2^ against ALL reflections. The weighted R-factor wR and goodness of fit S are based on F^2^, conventional R-factors R are based on F, with F set to zero for negative F^2^. The threshold expression of F^2^ > 2sigma(F^2^) is used only for calculating R-factors(gt) etc. and is not relevant to the choice of reflections for refinement. R-factors based on F^2^ are statistically about twice as large as those based on F, and R- factors based on ALL data will be even larger.

### Fractional atomic coordinates and isotropic or equivalent isotropic displacement parameters (Å^2^)

**Table:**

	*x*	*y*	*z*	*U*_iso_*/*U*_eq_	Occ. (<1)
Ag1	0.2324 (8)	0.3717 (4)	0.01361 (19)	0.0468 (5)	0.73 (3)
O2	0.1974 (4)	0.6015 (2)	0.04076 (18)	0.0513 (6)	
O1	0.3266 (4)	0.6125 (2)	−0.06037 (17)	0.0541 (7)	
N1	0.2472 (3)	1.1389 (2)	0.00461 (13)	0.0269 (5)	
C5	−0.1045 (4)	0.9302 (3)	−0.22018 (19)	0.0364 (7)	
H5	−0.1813	0.8855	−0.2717	0.044*	
C4	0.0109 (4)	0.8485 (3)	−0.15332 (19)	0.0327 (7)	
H4	0.0094	0.7481	−0.1591	0.039*	
C6	−0.1090 (4)	1.0819 (3)	−0.2124 (2)	0.0357 (7)	
H6	−0.1908	1.1366	−0.2583	0.043*	
C7	0.0054 (4)	1.1492 (3)	−0.13802 (19)	0.0330 (7)	
H7	0.0004	1.2495	−0.1336	0.040*	
C8	0.1322 (4)	1.0684 (3)	−0.06708 (17)	0.0250 (6)	
C3	0.1345 (4)	0.9134 (3)	−0.07441 (18)	0.0251 (6)	
C2	0.2579 (4)	0.8336 (3)	−0.00469 (17)	0.0269 (6)	
C1	0.2628 (4)	0.6678 (3)	−0.00950 (19)	0.0324 (7)	
C14	0.3754 (4)	0.9067 (3)	0.07046 (18)	0.0255 (6)	
C13	0.5050 (4)	0.8332 (3)	0.14539 (19)	0.0338 (7)	
H13	0.5140	0.7326	0.1451	0.041*	
C12	0.6146 (4)	0.9089 (3)	0.21674 (19)	0.0362 (7)	
H12	0.6975	0.8596	0.2650	0.043*	
C11	0.6044 (4)	1.0612 (3)	0.21852 (19)	0.0361 (7)	
H11	0.6798	1.1116	0.2681	0.043*	
C10	0.4860 (4)	1.1349 (3)	0.14873 (19)	0.0332 (7)	
H10	0.4828	1.2356	0.1507	0.040*	
C9	0.3668 (4)	1.0616 (3)	0.07275 (17)	0.0255 (6)	
Ag1'	0.270 (2)	0.3775 (9)	0.0018 (12)	0.058 (2)	0.27 (3)

### Atomic displacement parameters (Å^2^)

**Table:**

	*U*^11^	*U*^22^	*U*^33^	*U*^12^	*U*^13^	*U*^23^
Ag1	0.0732 (12)	0.0133 (4)	0.0485 (6)	−0.0002 (5)	0.0160 (5)	−0.0003 (3)
O2	0.0680 (17)	0.0186 (12)	0.0686 (16)	−0.0030 (11)	0.0265 (14)	0.0051 (11)
O1	0.0806 (18)	0.0260 (12)	0.0586 (15)	0.0118 (12)	0.0288 (14)	−0.0036 (11)
N1	0.0321 (13)	0.0161 (12)	0.0316 (13)	0.0028 (11)	0.0108 (11)	−0.0014 (10)
C5	0.0356 (18)	0.0374 (18)	0.0295 (15)	−0.0038 (14)	0.0040 (13)	−0.0056 (13)
C4	0.0328 (16)	0.0243 (16)	0.0353 (15)	−0.0014 (13)	0.0055 (13)	−0.0056 (12)
C6	0.0312 (17)	0.0395 (18)	0.0321 (16)	0.0054 (14)	0.0065 (13)	0.0085 (13)
C7	0.0373 (17)	0.0207 (15)	0.0388 (16)	0.0062 (13)	0.0114 (13)	0.0074 (12)
C8	0.0275 (15)	0.0196 (13)	0.0272 (14)	0.0015 (12)	0.0091 (12)	0.0007 (11)
C3	0.0273 (15)	0.0182 (13)	0.0286 (14)	−0.0022 (11)	0.0088 (12)	−0.0011 (11)
C2	0.0296 (15)	0.0170 (14)	0.0336 (16)	−0.0001 (11)	0.0108 (13)	0.0015 (11)
C1	0.0323 (16)	0.0229 (16)	0.0328 (16)	0.0025 (13)	0.0011 (13)	0.0004 (12)
C14	0.0266 (15)	0.0199 (14)	0.0285 (14)	−0.0023 (11)	0.0084 (12)	0.0004 (11)
C13	0.0359 (17)	0.0240 (15)	0.0360 (16)	0.0012 (13)	0.0068 (13)	0.0052 (12)
C12	0.0329 (17)	0.0377 (18)	0.0309 (15)	0.0023 (14)	0.0034 (13)	0.0060 (13)
C11	0.0337 (17)	0.0379 (18)	0.0313 (16)	−0.0059 (14)	0.0054 (13)	−0.0053 (14)
C10	0.0375 (17)	0.0240 (15)	0.0359 (15)	−0.0061 (14)	0.0110 (13)	−0.0064 (13)
C9	0.0288 (15)	0.0183 (13)	0.0293 (14)	−0.0025 (12)	0.0105 (12)	−0.0011 (12)
Ag1'	0.074 (3)	0.0130 (7)	0.072 (4)	−0.0051 (13)	0.009 (2)	0.0048 (15)

### Geometric parameters (Å, °)

**Table:**

Ag1—N1^i^	2.158 (4)	C7—C8	1.420 (4)
Ag1—O2	2.201 (4)	C7—H7	0.9300
O2—C1	1.266 (4)	C8—C3	1.435 (4)
O2—Ag1'	2.290 (15)	C3—C2	1.394 (4)
O1—C1	1.220 (4)	C2—C14	1.402 (4)
O1—Ag1'	2.499 (14)	C2—C1	1.532 (4)
N1—C8	1.348 (3)	C14—C9	1.432 (4)
N1—C9	1.356 (3)	C14—C13	1.433 (4)
N1—Ag1^ii^	2.158 (4)	C13—C12	1.356 (4)
N1—Ag1'^ii^	2.208 (8)	C13—H13	0.9300
C5—C4	1.355 (4)	C12—C11	1.408 (4)
C5—C6	1.406 (4)	C12—H12	0.9300
C5—H5	0.9300	C11—C10	1.352 (4)
C4—C3	1.424 (4)	C11—H11	0.9300
C4—H4	0.9300	C10—C9	1.416 (4)
C6—C7	1.362 (4)	C10—H10	0.9300
C6—H6	0.9300	Ag1'—N1^i^	2.208 (8)
			
N1^i^—Ag1—O2	170.0 (3)	C3—C2—C14	119.3 (3)
C1—O2—Ag1	103.2 (2)	C3—C2—C1	120.4 (2)
C1—O2—Ag1'	93.5 (6)	C14—C2—C1	120.4 (2)
C1—O1—Ag1'	85.0 (6)	O1—C1—O2	126.4 (3)
C8—N1—C9	119.4 (2)	O1—C1—C2	118.4 (3)
C8—N1—Ag1^ii^	120.5 (2)	O2—C1—C2	115.2 (3)
C9—N1—Ag1^ii^	120.12 (19)	C2—C14—C9	118.9 (2)
C8—N1—Ag1'^ii^	119.5 (4)	C2—C14—C13	122.9 (3)
C9—N1—Ag1'^ii^	120.5 (3)	C9—C14—C13	118.2 (3)
C4—C5—C6	120.6 (3)	C12—C13—C14	120.6 (3)
C4—C5—H5	119.7	C12—C13—H13	119.7
C6—C5—H5	119.7	C14—C13—H13	119.7
C5—C4—C3	121.2 (3)	C13—C12—C11	120.7 (3)
C5—C4—H4	119.4	C13—C12—H12	119.6
C3—C4—H4	119.4	C11—C12—H12	119.6
C7—C6—C5	120.4 (3)	C10—C11—C12	120.5 (3)
C7—C6—H6	119.8	C10—C11—H11	119.7
C5—C6—H6	119.8	C12—C11—H11	119.7
C6—C7—C8	120.9 (3)	C11—C10—C9	121.3 (3)
C6—C7—H7	119.5	C11—C10—H10	119.4
C8—C7—H7	119.5	C9—C10—H10	119.4
N1—C8—C7	119.4 (2)	N1—C9—C10	119.7 (2)
N1—C8—C3	122.0 (2)	N1—C9—C14	121.7 (2)
C7—C8—C3	118.6 (3)	C10—C9—C14	118.6 (3)
C2—C3—C4	123.1 (3)	N1^i^—Ag1'—O2	149.7 (13)
C2—C3—C8	118.8 (2)	N1^i^—Ag1'—O1	154.1 (12)
C4—C3—C8	118.1 (3)	O2—Ag1'—O1	55.0 (2)

Symmetry codes: (i) *x*, *y*−1, *z*; (ii) *x*, *y*+1, *z*.
